# DJ-1 and SOD1 Act Independently in the Protection against Anoxia in *Drosophila melanogaster*

**DOI:** 10.3390/antiox11081527

**Published:** 2022-08-05

**Authors:** Federica De Lazzari, Francesco Agostini, Davide Doni, Sandro Malacrida, Mauro A. Zordan, Paola Costantini, Luigi Bubacco, Federica Sandrelli, Marco Bisaglia

**Affiliations:** 1 Department of Biology, University of Padova, Via Ugo Bassi 58/B, 35131 Padova, Italy; 2 Medical Research Council, Mitochondria Biology Unit, University of Cambridge, Cambridge Biomedical Campus, Cambridge CB2 0XY, UK; 3 Institute of Mountain Emergency Medicine, Eurac Research, 39100 Bolzano, Italy; 4 Study Center for Neurodegeneration (CESNE), 35100 Padova, Italy

**Keywords:** amyotrophic lateral sclerosis, DJ-1, *Drosophila melanogaster*, Parkinson’s disease, SOD1

## Abstract

Redox homeostasis is a vital process the maintenance of which is assured by the presence of numerous antioxidant small molecules and enzymes and the alteration of which is involved in many pathologies, including several neurodegenerative disorders. Among the different enzymes involved in the antioxidant response, SOD1 and DJ-1 have both been associated with the pathogenesis of amyotrophic lateral sclerosis and Parkinson’s disease, suggesting a possible interplay in their mechanism of action. Copper deficiency in the SOD1-active site has been proposed as a central determinant in SOD1-related neurodegeneration. SOD1 maturation mainly relies on the presence of the protein copper chaperone for SOD1 (CCS), but a CCS-independent alternative pathway also exists and functions under anaerobic conditions. To explore the possible involvement of DJ-1 in such a pathway in vivo, we exposed *Drosophila melanogaster* to anoxia and evaluated the effect of DJ-1 on fly survival and SOD1 levels, in the presence or absence of CCS. Loss of DJ-1 negatively affects the fly response to the anoxic treatment, but our data indicate that the protective activity of DJ-1 is independent of SOD1 in *Drosophila*, indicating that the two proteins may act in different pathways.

## 1. Introduction

Superoxide dismutase 1 (SOD1) is one of the most important enzymes involved in the control of cellular redox homeostasis, owing to its ability to catalyze the dismutation of superoxide anions to hydrogen peroxide and molecular oxygen. SOD1 is a 32 kDa homodimeric metalloenzyme, which is mainly found in the cytosol, although it is also present in the nucleus and mitochondrial intermembrane space [1,2]. Each subunit is built upon an eight-stranded β-barrel and comprises two functionally important loops, called the electrostatic loop and the metal-binding loop, respectively, which play roles in protein folding and activity [1,2]. Each monomer incorporates one copper and one zinc ion in close enough proximity to share an imidazole ligand [1,2]. The zinc ion exerts a structural role, whereas the copper ion is the core of the enzymatic activity of the protein. The metal ions both contribute to the stability of the mature enzyme, which is further enhanced by an intramolecular disulfide bridge between the residues Cys57 and Cys146 [1].

After the discovery, in 1993 that mutations in the *SOD1* gene are involved in familial forms of amyotrophic lateral sclerosis (ALS), research concerning the physiopathology of the protein strongly increased. Since then, approximately 200 *SOD1* gene modifications have been described [1,2]. Pathogenic mutations are distributed throughout the entire sequence of the protein and all of them accentuate structural instability of the metal-free SOD1 (apo-SOD1) and promote the accumulation of disordered immature SOD1 conformers, which leads to the formation of intracellular aggregates [1,2]. Interestingly, data obtained in ALS mouse models, incorporating the pathological G37R or G93A SOD1 mutations, suggested that the copper content rather than the amino-acid mutations per se isi a greater determinant in motor neuron death and the ALS-like phenotype [3,4,5].

In addition to a prominent role exerted by SOD1 in the pathogenesis of ALS, a recent work reported an accumulation of abnormal deposits of SOD1 also in idiopathic Parkinson’s disease (PD) brains, strictly mirroring the pattern of neuronal loss observed in the disease [6]. Based on their results, the investigators proposed a model in which copper deficiency was associated with a reduction in the metal loading into the active site. As a consequence, apo-SOD1 becomes less stable, accumulates within aggregates, and loses its ability to protect neurons from oxidative damage [6,7,8]. 

Although both ALS and PD are complex pathologies, caused by the interplay of multiple genetic and environmental factors, the “metalation” status of SOD1 might be one of the key pathological determinants in both SOD1-related familial forms of ALS and sporadic PD. The copper acquisition has been shown to rely on a dedicated protein, referred to as copper chaperone for SOD1 (CCS) [9], through an oxygen-dependent pathway [10]. CCS is composed of three distinct domains, i.e., D1, D2, and D3. The first domain contains the copper-binding motif “MxCxxC”, which seems to be responsible for the acquisition of the metal ion from the plasma membrane copper transporter 1 (Ctr1) and for its delivery to SOD1. The D2 domain shares high homology with SOD1 and is involved in heterodimer formation. The last domain contains a CxC copper-binding motif that has been proposed to deliver copper into the SOD1 active site in an alternative way with respect to domain D1 [11]. 

Although SOD1 maturation mainly occurs via the CCS-dependent pathway, an alternative mechanism for copper insertion is known to exist and accounts for around 15% of SOD1 activity [12]. Moreover, the CCS-independent pathway is able to activate SOD1 even under anaerobic conditions [10]. In this frame, we and others demonstrated, in vitro, the ability of the protein DJ-1 to bind copper [13,14,15] and to interact with and activate SOD1, through copper transfer [15,16], suggesting a possible involvement of DJ-1 in the CCS-independent SOD1 maturation pathway. 

DJ-1 is a multifunctional protein implicated in oxidative stress responses, even though its specific role is still controversial [17,18,19]. Several different *DJ-1* gene mutations have been associated with familial autosomal recessive forms of PD [19,20]. Moreover, two independent studies have associated mutations in the *DJ-1* gene with ALS [21,22] and altered DJ-1 protein levels have been detected in cerebrospinal fluid, spinal cord, and motor cortex sections of ALS-affected patients [23,24].

Given the involvement of both SOD1 and DJ-1 in ALS and PD, and considering the purported participation of DJ-1 in the SOD1 maturation pathway, this work aimed to evaluate in vivo, using *Drosophila melanogaster* as an animal model, whether the antioxidant properties of DJ-1 were dependent on the presence of SOD1.

## 2. Materials and Methods

Drosophila Strains and Culture Maintenance—Flies were raised on agar, cornmeal, and yeast food, at 25 °C, under 70% relative humidity in 12-hour light/dark cycles. Only male flies were used in all experiments. The following strains were obtained from the Bloomington *Drosophila* Stock Center: *w^1118^* (#5905), *dj-1β*^Δ*93*^ (#33601), *Ccs^n29E^*/Cyo (#24755), *Sod1^x39^* (#24490), *Sod1^n1^* (#24492), *daughterless*-*Gal4* (#8641, da*Gal4*), and UAS-*dj-1β* (#33604). The *w^1118^* strain was used as a control line when analyzing *dj-1β*, *Ccs*, and *Sod1* mutant flies. 

Western blot analysis—To extract proteins, fly bodies were homogenized with a pestle in lysis buffer (50 mM Tris-HCl pH 7.5, 1% *v/v* Triton X-100, 1 mM EDTA, 1 mM sodium orthovanadate, 50 mM NaF, 10 mM p- glycerophosphate, 5 mM sodium pyrophosphate, and 270 mM sucrose), incubated on ice for 30 min, and then centrifuged at maximum speed for 30 min at 4 °C. Proteins were separated in 13% polyacrylamide SDS gels, and then transferred into nitrocellulose membranes (Bio-Rad), through a Trans-Blot^®^ Turbo™ TranSystem (Bio-Rad, Hercules, CA, USA). Membranes were blocked in Tris-buffered saline solution containing 0.1% Tween (TBS-T) and 5% *w*/*v* skimmed milk for 1 h at room temperature. After this step, membranes were incubated overnight at 4 °C with primary antibodies dissolved in 5% *w*/*v* skimmed milk in TBS-T. After incubation, membranes were washed 3 times for 10 min with TBS-T, and subsequently incubated with secondary antibodies conjugated with horseradish-peroxidase (HRP), diluted in 5% *w*/*v* skimmed milk in TBS-T for 1 h at room temperature. Finally, membranes were washed in TBS-T 3 times for 10 min. Proteins detection was performed using an ECL-Plus detection kit (GE Healthxare, Chicago, IL, USA ), and images were acquired using a VWR^®^ CHEMI Premium analyzer. Protein levels were quantified by densitometry using the ImageJ software (US National Institutes of Health). The primary antibodies were rabbit α-SOD1 (HPA001401, Sigma Prestige, 1:1000) and mouse α-actin (MAB1501, EDM Millipore 1:5000).

Survival experiments under anoxia—Adult males (1–3 days old) were collected under brief CO_2_ exposure and placed in fresh food vials 1 day before the experiment. On the day of the experiment, flies were incubated in an anaerobic glove box (MBRAUN MB-200B). Flies were kept under anoxia for up to 6 h, and, subsequently reintroduced into the normoxic atmosphere to monitor fly lethality.

Survival experiments under paraquat treatment—Groups of 20 flies (1–3 days old) were collected under brief CO_2_ exposure and placed in fresh food vials containing 1 mM paraquat. Paraquat sensitivity was determined by counting death events every day for 4 days.

Locomotion Assay—Groups of 20 flies (1–3 days old) were collected under brief CO_2_ exposure and placed in fresh food vials and the locomotion behavior was assessed the following day. When experiments were performed in the presence of paraquat, flies were transferred into new tubes every 2 days and the locomotion assays were performed after 7 days of treatment. The mobility of flies from each treatment group was assessed through a negative geotaxis climbing assay using a counter-current apparatus with 6 tubes in the lower frame and 5 in the upper frame. Flies were placed in the first plastic vial (1.5 cm diameter and 10 cm height) and gently tapped to the bottom. After 10 sec, the upper frame was moved to the right, and the flies that passed in the upper tubes during this period were transferred to the next lower tubes by gently tapping. This procedure was repeated 5 times. For each genotype, the climbing index was calculated using the following formula:

CI = [(#F5 × 5) + (#F4 × 4) + (#F3 × 3) + (#F2 × 2) + (#F1 × 1) + (#F0 × 0)/(#FT)](1)

where #Fn is the number of flies in the tube n (being 0 in the initial tube and 5 in the last tube) and #FT is the total number of flies.

Transmission electron microscopy *(TEM)*—To assess the mitochondrial morphology under basal conditions, adult males (1–3 days old) were fixed for 2 h in 0.1 M sodium cacodylate (pH 7.4), containing 2.5% glutaraldehyde and 2% paraformaldehyde, and then dissected to isolate thoraces. Briefly, the head, legs, and wings were removed with forceps, taking care to remove the gut as well. Subsequently, samples were incubated with a solution of 1% tannic acid for 1 h at room temperature, and then post-fixed with 1% osmium tetroxide in 0.1 M sodium cacodylate buffer for 1 h at 4 °C. After three water washes, samples were dehydrated in a graded ethanol series and embedded in an epoxy resin (Sigma-Aldrich). Ultrathin sections (60–70 nm) were obtained with an Ultrotome V (LKB) ultramicrotome, counterstained with uranyl acetate and lead citrate, and viewed using a Tecnai G2 (FEI) transmission electron microscope, operating at 100 kV. Images were captured with a Veleta (Olympus Soft Imaging System, Muenster, Germany) digital camera.

Statistical analysis—Data were collected from at least three independent experiments. Graphs were produced and statistical analyses were performed by using GraphPad Prism 9 software. One- or two-way ANOVA, followed by Tukey’s multiple comparisons post hoc test, was used for grouped comparisons. Survival analysis was performed by Mantel–Cox log-rank test. *p*-values <0.05 were considered to be significant.

## 3. Results

### 3.1. Ccs-Dependent and Ccs-Independent Sod1 Maturation Modulate D. melanogaster Life Expectancy, and Capability to Cope with Oxidative Stress Conditions

The *D. melanogaster* genome encodes homologs of the human proteins SOD1 and CCS, referred to as Sod1 and Ccs, respectively, and the CCS-dependent SOD1 maturation pathway has been described to be conserved in humans and flies [25]. More specifically, the physiological role of Ccs has been investigated through the generation of a *Ccs* null line (*Ccs^n29E^*), carrying a 1907 bp deletion at the *Ccs* locus level, which has been characterized long ago [25]. The strain shows a reduced lifespan and high sensitivity to oxidative conditions. Moreover, these effects appear to be derived from a reduced amount of functional Sod1, as the loss of Ccs affects both the levels and activity of Sod1. In this work, we confirmed some of the previously described effects induced by the depletion of Ccs, and we further characterized the *Ccs^n29E^* strain, by comparing it with the control line *w^1118^* and with a *Sod1* mutant. To this aim, we first tested the *Sod1^x39^* strain, characterized by a 395 bp deletion in the *Sod1* gene [26]. However, as the *Sod1* deletion was lethal in homozygosis, we moved to the *Sod1^n1^* strain bearing the G49S mutation that affects the formation of hydrogen bonds at the dimer interface. The G49S substitution makes the protein much more unstable as compared with the wild-type Sod1 form [26], but allows the development of alive homozygous adults, most probably because of a very small amount of active protein that is still present in these flies.

**Figure 1:**
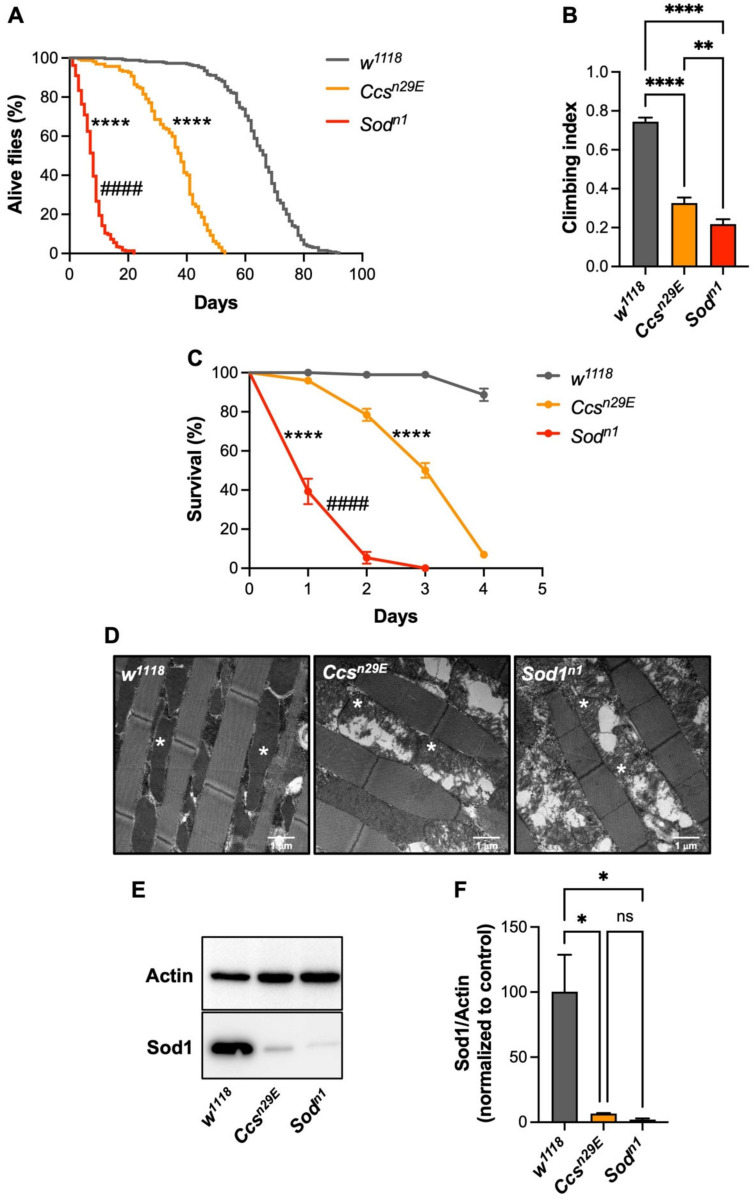
*Ccs^n29E^* null flies show milder phenotypes than *Sod1^n1^* mutants: (**A**) Survival analysis. *Ccs^n29E^* mutants lived significantly less than *w^1118^* controls but longer than *Sod1^n1^* flies (Mantel–Cox log-rank test: w^1118^ vs. Ccs^n29E^ (****), *w^1118^* vs. *Sod1^n1^* (****), *Ccs^n29E^* vs. *Sod1^n1^* (####), *p* < 0.0001 for all comparisons. N: 162 *Ccs^n29E^*, 288 *Sod1^n1^*, and 260 *w^1118^*); (**B**) climbing activity (mean ± SEM). Analysis of variance and post-hoc tests indicate significant locomotor impairments in *Ccs^n29E^* and *Sod1^n1^* flies as compared with *w^1118^* controls, with *Sod1^n1^* mutants showing the strongest phenotype (one-way ANOVA F_2, 516_ = 123.7 *p* < 0.0001; Tukey’s multiple comparisons test: *w^1118^* vs. *Ccs^n29E^* (****) and *w^1118^* vs. *Sod1^n1^* (****), *p* < 0.001; *Ccs^n29E^* vs. *Sod1^n1^* (**), *p* = 0.0075. N: 178 *Ccs^n29E^*, 164 *Sod1^n1^*, and 177 *w^1118^*); (**C**) survival analysis under mild oxidative stress conditions (1 mM paraquat). *Ccs^n29E^* flies were significantly more and less sensitive as compared with *w^1118^* and *Sod1^n1^* flies, respectively (Mantel–Cox log-rank test: *w^1118^* vs. *Ccs^n29E^* (****), *w^1118^* vs. *Sod1^n1^* (****), *Ccs^n29E^* vs. *Sod1^n1^* (####), *p* < 0.0001 for all comparisons. N: 172 *Ccs^n29E^*, 56 *Sod1^n1^*, and 99 *w^1118^*); (**D**) representative TEM images of the mitochondrial morphology of *Ccs^n29E^*, *Sod1^n1^* mutants, and *w^1118^* controls. Asterisks in the pictures indicate representative mitochondria; (**E**) representative Western blot; (**F**) relative quantification of Sod1 levels (mean ± SEM) in *Ccs^n29E^*, *Sod1^n1^*^,^, and *w^1118^* flies; in (**F**), Sod1 levels are reported as the Sod1/Actin ratio, with Actin signal used as a loading control. *Ccs^n29E^* and *Sod1^n1^* flies showed a significantly lower Sod1 amount with respect to controls (one-way ANOVA: F_2, 6_ = 11.51, *p* = 0.0088; Tukey’s multiple comparisons test: *w^1118^* vs. *Ccs^n29E^* (*) and *w^1118^* vs. *Sod1^n1^* (*) *p* < 0.05; *Ccs^n29E^* vs. *Sod1^n1^* (ns, non-significant) *p* = 0.97. N = 3).

In agreement with previous results [25], when we evaluated the lifespan of *Ccs* null individuals, we observed premature mortality with respect to controls, with the median survival time (t_1/2_)that decreases from 67 to 38 days, although they lived significantly longer than *Sod1* mutants, which were characterized by a t_1/2_ of only 8 days. (Figure 1A).

Then, we compared the locomotor behavior of flies through a negative geotaxis-based climbing assay, observing that, with respect to the controls, both *Ccs^n29E^* and *Sod1^n1^* lines showed a strong locomotor impairment, with slightly more pronounced effects in the case of *Sod1* mutants (Figure 1B). As a further characterization, we analyzed the effects derived by increasing oxidative stress conditions through the addition of paraquat into the food. Paraquat is an herbicide whose toxicity is generally ascribed to the generation of oxidative stress conditions through the production of free radical species, including superoxide and hydroxyl radicals, both at the cytosolic and mitochondrial levels [27,28,29]. Since it was previously shown that in the presence of 2 mM paraquat, *Ccs^n29E^* flies display the same extreme hypersensitivity to the redox cycling agent as exhibited by the *Sod1^n1^* line, with less than 5% of survivors after 24 h [25], here, we used a lower concentration of paraquat and measured fly survival for a longer period (4 days). As expected, in the presence of 1 mM paraquat, the absence of Ccs induced a high sensitivity to oxidative conditions in contrast to control flies, whose survival was almost unaffected throughout the time course of the experiment. However, as for the lifespan and the climbing ability, the lack of Ccs produced milder effects than the loss of the Sod1 protein itself (Figure 1C). Given that oxidative stress and mitochondrial damage are often correlated, to further evaluate the consequences of the loss of Ccs, we evaluated the mitochondrial morphology of both *Ccs^n29E^* and *Sod1^n1^* mutants and negative controls. In contrast to *w^1118^* control flies and similarly to *Sod1^n1^* mutants, the absence of Ccs in *Ccs^n29E^* individuals led to swollen and vacuolized mitochondria characterized by an altered ultrastructure. (Figure 1D). Therefore, the loss of either Ccs or Sod1 largely impacts mitochondrial homeostasis. Since it has been reported that upon Ccs depletion, the *Drosophila* Sod1 protein becomes unstable and its levels dramatically drop [25], we finally evaluated the amount of Sod1 in *Ccs* null flies, by immunoblotting. Consistent with the previous report, the loss of Ccs highly impairs Sod1 protein levels (Figure 1E), confirming that Sod1 becomes unstable in the absence of its copper chaperone. Nevertheless, we detected a higher residual amount of Sod1 in *Ccs* null flies with respect to *Sod1^n1^* mutants (Sod1 levels (mean ± SEM): 6.6 ± 0.5 and 2.1 ± 0.9 in *Ccs^n29E^* and *Sod1^n1^*, respectively) (Figure 1F), although the difference was not statistically significant, which might explain the less robust phenotypic effects in *Ccs^n29E^* as compared with *Sod1^n1^* flies. Overall, our results indicate that the absence of Ccs strongly affects the capability of flies to cope with oxidative stress conditions. Furthermore, taken together, our data confirm that also, in the *D. melanogaster* model, Sod1 maturation can be achieved through an alternative Ccs-independent pathway.

### 3.2. Drosophila dj-1β Participates in the Protection against Oxygen Deprivation without Affecting Sod1 Expression

In light of the results presented above, we were interested in assessing whether DJ-1 could be implicated in the CCS-independent SOD1 maturation. The *D. melanogaster* genome encodes two DJ-1 homologs, referred to as dj-1α and dj-1β, with a different pattern of expression, being dj-1α expression mainly restricted to the testis while dj-1β shows a ubiquitous expression profile. Double *dj-1α*; *dj-1β* knock-out animals display enhanced sensitivity to oxidative conditions [30]. Interestingly, this sensitivity has been demonstrated to depend on the loss of dj-1β, since single *dj-1β* null flies (*dj1β*^Δ^*^93^*) are as sensitive to oxidative stressors as double *dj-1α* and *dj-1β* knock-out flies, while *dj-1α* single knock-out individuals behave as control flies [30]. Moreover, it has been previously demonstrated that human DJ-1 can rescue *dj-1β* knock-out phenotypes when flies were treated with paraquat, underscoring the similarity between the human and fly proteins [31]. Consequentially, we focused on the protective effects of the dj-1β isoform. As the CCS-independent SOD1 maturation process does not require the presence of molecular oxygen, to investigate the possible involvement of dj-1β in this pathway, we first tested how the modulation of dj-1β expression levels affects fly survival by comparing *dj-1β* knock-out with *w^1118^* and *Ccs^n29E^* flies under anoxic conditions. With respect to mammals, fruit flies represent a valuable in vivo model for this kind of experiment as they are very resistant to anoxia for up to a few hours [32].

**Figure 2:**
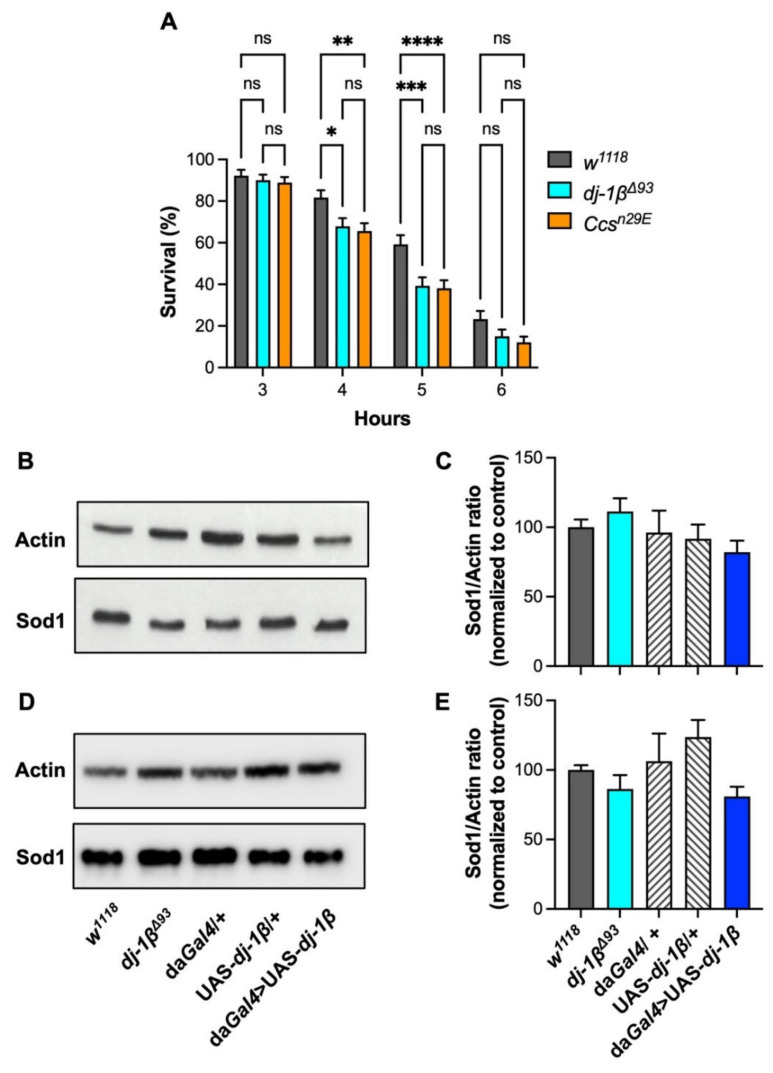
*dj-1β* protects against oxygen deprivation without affecting Sod1 expression: (**A**) Percentage of survivors (mean ± SEM) in *dj-1β*^Δ*93*^, *Ccs^n29E^*, and *w^1118^* control flies, after 3, 4, 5, and 6 h of anoxic treatment. *dj-1β*^Δ*93*^ and *Ccs^n29E^* flies showed significantly higher mortality as compared with *w^1118^* controls after 4 and 5 h of anoxia. Anoxia induced similar effects in both *dj-1β*^Δ*93*^ and *Ccs^n29E^* flies, at all time points (two-way ANOVA (time of treatment X genotype) F_6, 1578_ = 1.28, *p* = 0.263; time of treatment effect: F_3, 1578_ = 222.4, *p* < 0.0001; genotype effect: F_2, 1578_ = 13.83, *p* < 0.0001; in the graph ****, ***, **, *, and ns indicate *p* < 0.0001, < 0.001, < 0.01, < 0.05, and non-significant, in Tukey’s multiple comparisons post-hoc tests, see text for details); (**B**) representative Western blot and (**C**) relative quantification of Sod1 protein levels (mean ± SEM) under basal conditions, in *dj-1β* KO and *dj-1β*-overexpressing (da*Gal4* > UAS-*dj-1β*) flies as compared with controls (*w^1118^*, da*Gal4*/+ and UAS-*dj-1β*/+); in (**C**), Sod1 levels are reported as the Sod1/Actin ratio, with Actin signal used as a loading control. No significant differences in Sod1 protein signals were detected among genotypes (one-way ANOVA F_4, 23_ = 0.90, *p* = 0.483, N > 4 per genotype); (**D**) representative Western blot and (**E**) relative quantification of Sod1 protein levels (mean ± SEM) in *dj-1β* KO and *dj-1β*-overexpressing (da*Gal4* > UAS-*dj-1β*) flies as compared with controls (*w^1118^*, da*Gal4*/+, and UAS-*dj-1β*/+*),* after 7 days of exposure to 1 mM paraquat; in (**E**), Sod1 protein amounts are reported as the Sod1/Actin ratio, with Actin signal used as a loading control. No significant differences in Sod1 protein amounts were detected among genotypes (one-way ANOVA F_4, 17_ = 2.29 *p* = 0.101; N > 3 per genotype).

When flies were exposed to anoxia, they rapidly stopped moving and fell into a coma-like condition within a few minutes. Since the rescue of locomotor activities after the reintroduction into a normoxic atmosphere required several hours to be completed, we measured fly survival one day after treatment as a readout of the anoxic effect. Figure 2A shows the survival profiles of *dj1β*^Δ*93*^, *Ccs^n29E^*, and control flies kept under anoxic conditions for different periods (3, 4, 5, and 6 h). For each genotype, the percentage of surviving flies decreases with increasing time of treatment, indicating a direct correlation between the extent of the anoxia period and lethality. Moreover, while the percentages of survivors in *dj1β*^Δ*93*^ and *Ccs^n29E^* strains were similar to control individuals after 3 h of anoxic treatment, 4 and 5 h of anoxic exposure induced significantly higher mortality in both *dj1β*^Δ*93*^ and *Ccs^n29E^* as compared with the controls (Figure 2A). The difference among genotypes disappeared after 6 h of treatment, which determined the death of most of the flies in both *dj1β*^Δ*93*^ and *Ccs^n29E^*, as well as in control samples (Figure 2A), indicating that 6 h of anoxic exposure was too drastic to detect statistically significant differences among the three *Drosophila* genotypes. Interestingly, the effects observed under oxygen deprivation did not significantly differ between *dj1β*^Δ*93*^ and *Ccs^n29E^* strains (Figure 2A).

In mammals, DJ-1 has been reported to stimulate the nuclear translocation of the extracellular signal-regulated protein kinases 1 and 2 (ERK1/2), which phosphorylate ets like-1 protein (Elk1), a transcription factor involved in the expression of various antioxidant genes, including SOD1 [33]. Therefore, to assess whether the protective role of dj-1β observed during anoxic treatments was associated with its capability to promote the expression of Sod1, we measured Sod1 protein levels in *dj-1β* knock-out and control flies. As represented in Figure 2B,C, the absence of dj-1β does not affect the amount of protein. Additionally, Sod1 protein levels resulted similar in dj-1β overexpressing flies (da*Gal4* > UAS-*dj-1β*) as compared with their appropriate negative controls (da*Gal4*/+ and UAS-*dj-1β*/+) (Figure 2B,C), further ruling out a direct involvement of dj-1β in the modulation of Sod1 expression under our experimental conditions.

Since the most corroborated function of DJ-1 deals with its protective role against oxidative stress, we wondered whether the participation of dj-1β in the modulation of Sod1 expression could become relevant under oxidative conditions. However, simple discrimination between dead and alive individuals immediately after the anoxic incubation period was impossible, because of the coma-like state of alive flies. Moreover, during the long-time interval required by flies to completely recover after the anoxic treatment, protein levels could change. For these reasons, the analysis of Sod1 expression under anoxia was not informative and we adopted an alternative approach: we measured Sod1 protein levels after treatment with a sub-lethal concentration of paraquat (1 mM). Our data indicate that, also under oxidative conditions, dj-1β is unable to modulate Sod1 expression (Figure 2D,E), suggesting that the protective effects mediated by dj-1β do not depend on *Sod1* transcriptional activation.

### 3.3. The Overexpression of dj-1β Does Not Rescue the Effects Induced by Ccs Depletion

**Figure 3:**
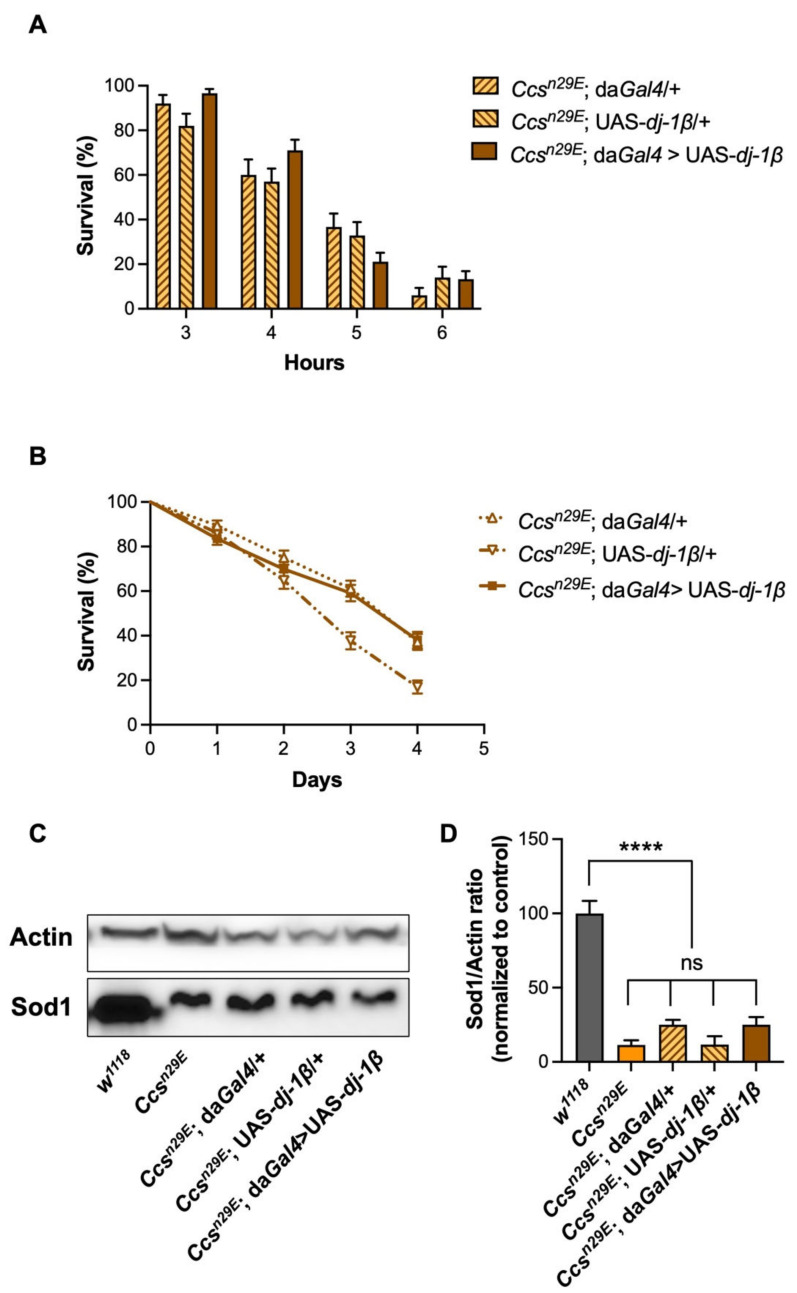
*dj-1β* overexpression in a *Ccs^n29E^* background does not rescue the effects induced by a Ccs depletion: (**A**) Percentage of survivors (mean ± SEM) in *Ccs^n29E^*, da*Gal4* > UAS-*dj-1β,* and relative controls (*Ccs^n29E^*, da*Gal4*/+ and Ccs^n29E^, UAS-*dj-1β*/+*),* after 3, 4, 5, and 6 h of anoxic treatment. Time of anoxic treatment significantly affected survival in all fly strains, with no differences among genotypes (two-way ANOVA (time of treatment X genotype) F_6, 798_ = 2.78, *p* = 0.011; time of treatment effect: F_3, 798_ = 144.1, *p* < 0.0001; genotype effect: F_2, 798_ = 0.76, *p* = 0.47, non-significant). For each time of treatment, 50–90 males per genotype were analyzed; (**B**) survival analysis under mild oxidative stress conditions (1 mM paraquat) in *Ccs^n29E^*; da*Gal4* > UAS-*dj-1β,* and relative controls (*Ccs^n29E^*, da*Gal4*/+ and Ccs^n29E^, UAS-*dj-1β*/+*).* The *Ccs^n29E^*; da*Gal4* > UAS-*dj-1β* survival profile was intermediate between the two controls, indicating the differences were not due to a *dj-1β* overexpression in a *Ccs* null background (Mantel–Cox log-rank test: *Ccs^n29E^*; da*Gal4* > UAS-*dj-1β* vs. *Ccs^n29E^*; da*Gal4*/+: *p* = 0.8; *Ccs^n29E^*; da*Gal4* > UAS-*dj-1β* vs. Ccs^n29E^; UAS-*dj-1β*/+: *p* = <0.0001; *Ccs^n29E^*; da*Gal4*/+ vs. Ccs^n29E^; UAS-*dj-1β*/+: *p* <0.0001); (**C**) Representative Western blot and (**D**) relative quantification of Sod1 levels (mean ± SEM) in *Ccs^n29E^*; da*Gal4* > UAS-*dj-1β* and control flies; in (**D**), Sod1 levels are reported as the Sod1/Actin ratio, with Actin signal used as a loading control. Sod1 levels in *Ccs^n29E^*; da*Gal4* > UAS-*dj-1β* flies were significantly lower from that of *w^1118^* controls but similar to those of *Ccs*^n29E^ null flies and *Ccs^n29E^*; da*Gal4*/+ or Ccs^n29E^; UAS-*dj-1β*/+ controls (one-way ANOVA: F_4, 10_ = 46.1, *p* < 0.0001; Tukey’s multiple comparisons test: *w^1118^* vs. all other genotypes *p* < 0.0001 (****), *Ccs^n29E^*, all other comparisons, *p* ≥ 0.4, ns. N = 3).

After having characterized several experimental readouts related to the loss of the Ccs protein, we investigated the potential complementarity between dj-1β and Sod1 in the antioxidant response, by either using *dj-1β* knock-out flies or by ubiquitously overexpressing dj-1β in a *Ccs* null background. First, through a series of standard crosses, we produced a *Ccs^n29E^*/Cyo; *dj-1β*^Δ*93*^/*dj-1β*^Δ*93*^ line with the aim of characterizing the *Ccs*; *dj-1β* double knockout individuals. Interestingly, the genetic deletion of both *Ccs* and *dj-1β* was lethal, as homozygous flies did not eclose, coherently with the initial hypothesis that both proteins could participate in Sod1 maturation in two independent ways. To evaluate this indication, we assessed the effects of dj-1β overexpression in the absence of Ccs, using *Ccs^n29E^*; da*Gal4* > UAS-*dj-1β* flies, and relative controls (*Ccs^n29E^*; da*Gal4*/+ and *Ccs^n29E^*; UAS-*dj-1β*/+*)*. Considering the involvement of dj-1β in the antioxidant response and the existence of a Ccs-independent Sod1 maturation pathway which does not rely on the presence of oxygen, we carried out survival experiments after anoxic treatments or in paraquat-induced oxidative conditions. Unexpectedly, in both cases, the results did not support a complementary role for dj-1β in the transfer of copper into the Sod1 active site. In fact, under oxygen deprivation, the survival of dj-1β overexpressing flies was comparable to those of both parental lines at each time point considered (Figure 3A).

Moreover, the effects induced by the presence of paraquat were very similar between controls and dj-1β-overexpressing flies (Figure 3B). Since, as described above, upon Ccs depletion, *Drosophila* Sod1 becomes unstable and its levels drop dramatically, we used Sod1 protein levels as an experimental readout to further evaluate the possible participation of dj-1β in the Ccs-independent Sod1 maturation pathway. As represented in Figure 3C, the expression levels of Sod1 in each strain presenting a *Ccs* null background were very low as comparison with *w^1118^* flies, even in the presence of dj-1β overexpression, excluding, once again, the involvement of dj-1β in the process that leads to the accumulation of the mature form of Sod1.

## 4. Discussion

Currently, reactive oxygen species (ROS) have been recognized to play important functions as endogenous mediators in several signaling pathways. However, ROS are extremely reactive molecules and, if their concentration rises above physiological levels, they can exert deleterious effects. Accordingly, numerous neurodegenerative disorders, including ALS and PD, are characterized by ROS-associated oxidative damage. It follows that the fine-tuning between ROS generation and their elimination is essential for cell survival. Among the different enzymes involved in the antioxidant response, in this work, we focused on SOD1 and DJ-1 and their possible interplay, since both have been associated with the pathogenesis of ALS and PD.

While the enzymatic function of SOD1 is very well characterized, being the enzyme involved in the dismutation of superoxide radicals, much less is known about the precise mechanism through which DJ-1 exerts its antioxidant function. Interestingly, independent lines of research have suggested a possible involvement of DJ-1 in binding copper ions and protecting against copper-induced cytotoxicity [13,14,15], although contrasting results have also been reported [34]. Moreover, based on in vitro and cellular indications, we and others have proposed the potential participation of DJ-1 in the SOD1 activation [15,16,35,36], suggesting the possibility that the antioxidant activity of DJ-1 is mediated by SOD1.

In this work, this hypothesis has been explored using *D. melanogaster* as an in vivo model. Fruit flies possess orthologs of the human protein objects of this study that share similar functional properties with their human counterparts. Consistent with previously published data [25], our results suggest that the presence of Sod1 plays a protective role for adult flies, especially in the presence of clear oxidative insults, since the amount of protein is directly correlated with both lifespan and fly survival in the presence of paraquat. More importantly, the phenotypes observed in *Ccs* null mutants support the presence, as in humans, of a Ccs-independent Sod1 maturation pathway.

As the Ccs-independent pathway does not rely on the presence of oxygen, then, we decided to carry out experiments under anaerobic conditions. First, we demonstrated that the lack of Ccs makes flies more sensitive to oxygen depletion. This observation can be explained considering that, in the absence of Ccs, the Sod1 protein does not accumulate in its active form. Unexpectedly, however, in the *Cc*s null background, the overexpression of dj-1β was unable to provide protection under anoxia and similar results were also observed in the presence of paraquat, a herbicide that has been demonstrated to increase the cellular production of ROS. Several hypotheses might explain the discrepancy between the results presented in this work and previous studies from our and other laboratories, which suggested a role for DJ-1 in the CCS-independent SOD1 maturation pathway.

One possible explanation could be linked to the fact that, in *Drosophila,* the absence of Ccs makes Sod1 highly unstable, actually subtracting Sod1 to the action of dj-1β. Elevated Sod1 instability in *Ccs* null mutant flies has been reported in a previous study [25] and validated in our work. Moreover, such behavior has been confirmed by expressing *Drosophila* Sod1 in a yeast strain depleted of the endogenous CCS but was not observed with yeast and human SOD1 [25]. The authors also demonstrated that Sod1 stabilization was dependent on copper transfer and/or disulfide oxidation and proposed that, in *Drosophila*, Ccs afforded stability to Sod1 by activating the enzyme through copper insertion and/or disulfide oxidation [25]. In this frame, it is plausible that DJ-1 requires the presence of SOD1 in a folded state to transfer the copper ion and activate the enzyme.

However, considering that DJ-1 seems to possess redundant cellular functions and that only mild phenotypes are observed in DJ-1 knock-out animal models, a further possibility to explain our results is that the protective role of dj-1β could become particularly important only under mildly stressful situations. In other words, the strong phenotypes caused by the loss of Ccs could be too strong to allow the detection of the limited protective effects mediated by dj-1β. In agreement with this hypothesis, it has recently been shown that while the loss of DJ-1 makes cells more sensitive to methylglyoxal-associated glycation, its overexpression does not improve cellular viability against the toxicity of exogenously added methylglyoxal [37].

On the contrary, we consider it unlikely that the discrepancy between our in vivo results and previously published in vitro and cellular data is due to an evolutionary functional divergence between *Drosophila* dj-1β and human DJ-1. This hypothesis could find support in the fact that in human DJ-1, two cysteine residues, namely Cys53 and Cys106, have been described as fundamental in forming two different copper-binding sites [14,15], while *Drosophila* dj-1β lacks the cysteine residue corresponding to human DJ-1 Cys53. However, dj-1β possesses the Cys104 residue, corresponding to human DJ-1 Cys106, which has been shown to be a key site allowing the transfer of the metal ion to SOD1 in vitro [15]. Moreover, the Cys53 residue is also absent in the *Arabidopsis thaliana* DJ-1 homolog, whose role in SOD1 maturation was described for the first time [16].

Finally, we also tend to exclude that our in vivo data are an indication that, in *Drosophila,* dj-1β works upstream from Ccs in the same cellular pathway, in agreement with the hypothesis that a still unknown protein is responsible for the transfer of intracellular copper from glutathione to CCS [38]. In fact, while both *Ccs* and *dj-1β* single mutants reach the adult stage, the *Ccs*; *dj-1β* double knock-out is lethal, therefore, suggesting that Ccs and dj-1β proteins exert their role in distinct protective pathways.

Notably, our results indicate that the absence of dj-1β affects adult fly survival under oxygen depletion, in line with a purported protective role of DJ-1 against hypoxia injury [18]. Remarkably, the effects observed in *dj-1β* knock-out mutants do not seem to rely on the Ccs-dependent Sod1 maturation pathway, as *dj-1β* knock-out flies showed an accumulation of Sod1 similar to controls. Additionally, the overexpression of dj-1β did not alter Sod1 expression, either at the RNA or protein levels, again indicating that the protective function of dj-1β was independent of Sod1.

**Figure 4:**
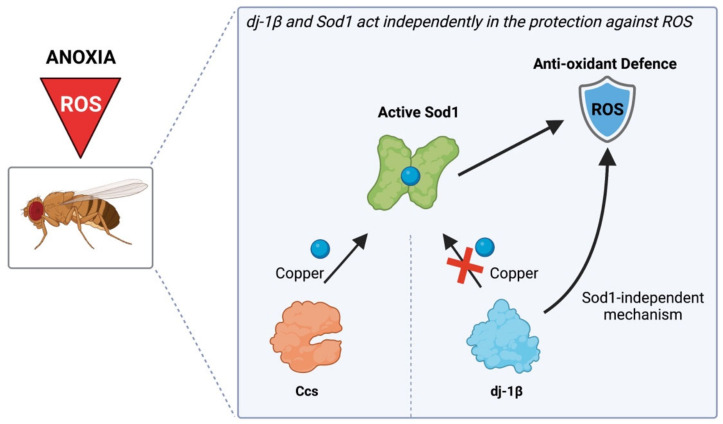
Proposed model describing the independent actions of Sod1 and dj-1β in the protection against anoxia. In *Drosophila melanogaster*, Ccs is responsible for copper loading into the Sod1 active site through the so-called Ccs-dependent Sod1 maturation pathway. The loss of Ccs increases the susceptibility of flies to oxidative stress. dj-1β does not participate in the Ccs-independent Sod1 maturation pathway, as in a *Ccs* null genetic background, dj-1β overexpression does not induce any protection. The loss of dj-1β increases the susceptibility to oxidative stress without affecting the Ccs-related Sod1 maturation pathway, as Sod1 levels in *dj-1β* knock-out flies are similar to controls. Moreover, dj-1β protection appears independent from Sod1 as the modulation of dj-1β expression does not affect Sod1 levels either under basal conditions or in the presence of oxidative stress (created with BioRender.com, accessed on 7 July 2022).

In conclusion, as summarized in Figure 4, the picture arising from the data presented here is that dj-1β and Sod1 exert their antioxidant activity through two distinct mechanisms, and further research is still required to unmask the precise molecular mechanisms underlying the protective role of DJ-1.

## Data Availability

Not applicable.
